# Novel *in situ* gelling vaginal sponges of sildenafil citrate-based cubosomes for uterine targeting

**DOI:** 10.1080/10717544.2018.1477858

**Published:** 2018-06-05

**Authors:** Heba M. Aboud, Amira H. Hassan, Adel A. Ali, Abdel-Razik H. Abdel-Razik

**Affiliations:** a Department of Pharmaceutics, Faculty of Pharmacy, Beni-Suef University, Beni-Suef, Egypt;; b Department of Histology, Faculty of Veterinary Medicine, Beni-Suef University, Beni-Suef, Egypt

**Keywords:** Sildenafil citrate, cubosomes, *in-situ* gelling sponges, uterine targeting, pharmacokinetic study

## Abstract

Sildenafil citrate (SIL), a type 5-specific phosphodiesterase inhibitor, demonstrates valuable results in the management of infertility in women; however, the absence of vaginal dosage form in addition to the associated oral adverse effects minimize its clinical performance. The present study is concerned with SIL uterine targeting following intravaginal administration *via* optimization of cubosomal *in situ* gelling sponges (CIS). An emulsification method was employed for preparation of cubosomal dispersions incorporating glyceryl monooleate as a lipid phase and poloxamer 407 as a surfactant with or without polyvinyl alcohol as a stabilizer. Cubosomes were estimated regarding entrapment efficiency (EE%), particle size, and *in vitro* drug release. Chitosan (2% w/w) was incorporated into the optimum formulation and then lyophilized into small sponges. For the CIS, *in vivo* histopathological and pharmacokinetic studies were conducted on female Wistar rats and compared with intravaginal free SIL sponges (FIS) and oral SIL solution. SIL-loaded cubosomes showed EE% ranging between 32.15 and 72.01%, particle size in the range of 150.81–446.02 nm and sustained drug release over 8 h. Histopathological study revealed a significant enlargement in endometrial thickness with congestion and dilatation of endometrial blood vessels in intravaginal CIS compared to intravaginal FIS and oral-treated groups. The pharmacokinetic study demonstrated higher AUC_0–∞_ and *C*_max_ with oral administration compared to intravaginal CIS or intravaginal FIS indicating potential involvement of first uterine pass effect after intravaginal administration. Finally, intravaginal CIS could be considered as a promising platform for SIL uterine targeting with minimized systemic exposure and side effects.

## Introduction

The most popular intricacy of pregnancy is the recurrent spontaneous abortion (RSA), which is defined as the clinical deprivation of pregnancy for three or more times (El-Far et al., 2009). RSA is considered as a critical health complication for 2–5% of the reproducing couples all over the world. One of the promising treatment tools employed for RSA complication is *in vitro* fertilization (IVF) with inconstant outputs (Sher & Fisch, 2002; Takasaki et al., 2010). The incidence of pregnancy in IVF-treated patients has been associated with the endometrial thickness of more than 8 mm (Gonen & Casper, 1990; Sher et al., 1991). Indeed, abundant treatments have been proposed for improvement of the endometrial thickness such as aspirin in low dose and estrogens (Weckstein et al., 1997). Additionally, great attention has been paid to the enhancement of vasodilation of endometrial vasculature *via* amelioration of uterine blood flow for endometrial lining proliferation (Amit et al., 1998).

Sildenafil citrate (SIL), a type 5-specific phosphodiesterase inhibitor, has been documented for enhancement of uterine blood flow after intravaginal administration which in turn leads to fruitful pregnancy as a result of increased endometrial lining proliferation *via* estrogen induction (Sher & Fisch, 2000; Zinger et al., 2006; Villanueva-García et al., 2007; Jerzak et al., 2008; Dehghani Firouzabadi et al., 2013). Moreover, SIL is deemed as a valuable candidate for treatment of intrauterine growth retardation or uterine hypermotility in pregnant women (Sher & Fisch, 2000; Zinger et al., 2006; Villanueva-García et al., 2007). Meanwhile, oral SIL administration exhibits many side effects mainly manifest as headaches, flushing and hypotension as well as its probable low uterine (target) level which makes oral SIL treatment ineffective in pregnant women (Sher & Fisch, 2000, 2002). Most researches were performed using commercial oral SIL tablets due to the absence of SIL vaginal dosage form which necessities its administration for four times daily to attain an effective therapy (Sher & Fisch, 2002). This repeated administration is possibly attributed to fast tablet clearance through the vaginal fluids in addition to SIL exhibits minimal permeability and lipophilicity at vaginal pH (Wang et al., 2008). The aforementioned facts warrant the development of an adequate intravaginal delivery system for SIL uterine targeting for RSA treatment with the least systemic concentrations and adverse effects. Drug encapsulation in micro- or nanocarriers is one of the adopted modern approaches in vaginal drug delivery systems.

Cubosomes or cubic-phase nanoparticles are nanostructures consisted chiefly of amphiphilic polar lipid which forms micellar aggregations upon dissolving in water when used at a level above the critical micelle concentration. The created micelles are compelled to form cubic structure at higher concentrations (Milak & Zimmer, 2015). Also, heating is substantial to prepare that cubic crystalline liquid. At lower temperature, the hydrocarbons chains of the polar lipid melt while the polar heads stay robust and joined together with intense hydrogen bonds (Yang et al., 2004). In the liquid crystalline state, the conformation of the carbon–carbon bonds is altered from all-trans to the gauche (Garti et al., 2012). The intrinsic feature of the cubosomal structure is the amalgamation between the highly ordered planar layers and the disordered melted atoms. Glyceryl monooleate (GMO) is the polar lipid utilized in the preparation of cubosomes which has a polar head consisted of the glycerol part and a lipophilic moiety represented by the C18 chain possessing a unique double bond at C9 (Ganem-Quintanar et al., 2000).

Various appealing characters of GMO have been reported: (i) it subjects to lipolysis which makes the cubic phase biodegradable; (ii) oil soluble, water soluble, or amphiphilic drugs can be incorporated within its lipid or aqueous domains (Li et al., 2015) and (iii) suitable organization of the bicontinuous cubic phase and the controlled release manner renders it favorable and attractive for drug delivery (Gabr et al., 2017). Depending on varied periodic minimal surfaces of the lipid bilayer, three distinct inverted bicontinuous cubic phases might be recognized experimentally; the primitive cP (space groups Im3 m), the double diamond cD (space groups Pn3 m), and the gyroid cG (space groups Ia3d) (Kulkarni et al., 2017).

Although cubosomes are promising candidates for mucosal drug delivery, to the authors’ knowledge, the employment of this nanocarrier for the amelioration of vaginal drug delivery was attempted for propantheline bromide and oxybutynin hydrochloride only (Guo et al., 2010). However, no data involving *in vivo* experiment were reported. The current study investigates the potential of this nanocarrier for the uterine targeting of SIL with *in vivo* testing in female Wistar rats.

Unfortunately, intravaginal drug delivery has some limitations such as rapid loss of the applied formulation as a result of the influence of gravity and clearance by vaginal fluids leading to decreased drug efficiency (Woolfson et al., 2000). Hence, the development of new systems that can prolong the residence time of vaginal formulations is substantial to overcome this encumbrance.

In this study, *in situ* gelling sponges are evoked as an elegant substitute to conventional vaginal delivery systems like suppositories and gels. Mucoadhesive vaginal sponges based on water soluble polymers permit simple application and precise dosing as a result of their single and solid dose feature. They are designed to hydrate rapidly in the vaginal cavity into a mucoadhesive gel with extended permanence and prolonged drug release and without provoking foreign body sensation. The development of such pioneered drug delivery system needs only conventional techniques as freeze-drying (Werner et al., 2004).

Different SIL vaginal delivery systems, including the use of controlled release formulations (Değim et al., 2008), thermosensitive gels (Soliman et al., 2017), and liposomes (Refai et al., 2017) have been carried out for enhancing its therapeutic efficacy. To the best of our knowledge, this is the first study to explore the cubosomal *in situ* gelling sponges (CIS) as a potential solid nanocarrier surrogate for SIL uterine targeting.

In view of the above, the aim of this study is to prepare, optimize, and characterize SIL-loaded cubosomes, followed by converting the optimal cubosomal dispersion into *in situ* gelling sponge. Moreover, the *in vivo* behavior and the uterine targeting efficiency of the CIS were evaluated in female rats. Histopathological study was also included in the current investigation.

## Materials and methods

### Materials

Sildenafil citrate was received as a gift sample from Eipico (Cairo, Egypt), glyceryl monooleate, polyvinyl alcohol (molecular weight 12,000–13,000 Da and 87–89% degree of hydrolysis), chitosan (low molecular weight: 150,000 Da and 75–85% degree of acetylation), acetonitrile (HPLC grade), methanol (HPLC grade), diethyl ether (HPLC grade) were purchased from Sigma-Aldrich (St. Louis, MO). Dialysis bags with a molecular weight cut-off of 12,000 Da were purchased from SERVA Electrophoresis GmbH (Heidelberg, Germany). Triton X-100 was purchased from Loba Chemie (Mumbai, India). All other ingredients used were of analytical grade.

### Preparation of SIL-loaded cubosomes

Cubosomes were prepared according to the emulsification of monoglyceride/surfactant mixtures in water (Esposito et al., 2003). GMO was used as the lipid phase in the concentration of 5% w/w with respect to the total weight of the dispersion and poloxamer 407 (P-407) was used as a surfactant in a concentration range between 5 and 15% w/w with respect to the disperse phase. Polyvinyl alcohol (PVA) was used in addition to P-407 as a stabilizing agent for the dispersion, and it was added by solubilization at 80 °C in the aqueous phase in various concentrations (0%, 1%, 2.5% or 5% w/w with respect to the disperse phase). SIL (10 mg) was dissolved in the aqueous phase. Briefly, GMO and P-407 were melted in a thermostatically controlled water bath at 70 °C. Afterwards, the molten mixture was gently injected using an insulin syringe into a preheated aqueous phase at 70 °C and emulsified using an Ultra Turrax homogenizer (Ultra Turrax^®^ T 25 basic homogenizer, IKA, Staufen, Germany) at 8000 rpm for 10 min. The final dispersion was cooled and maintained at ambient temperature for further investigation.

### Statistical design of the study

A 4^2^ full-factorial experimental design was applied to explore the effects of different variables on the characteristics of cubosomal formulations using Design-Expert^®^ software (Version 7, Stat-Ease Inc., Minneapolis, MN). The independent variables were the concentration of P-407 (surface active agent) (*X*_1_) and PVA (stabilizer) (*X*_2_). The investigated responses were EE% (*Y*_1_), particle size (*Y*_2_), % SIL released from cubosomes in 8 h (*Y*_3_), as dependent variables. According to the statistical plan, P-407 concentrations were 5, 7.5, 10, and 15% and PVA concentrations were 0, 1, 2.5, and 5%. Composition of the developed cubosomal formulations is presented in Table 1.

**Table 1:** Experimental runs, independent variables, and measured responses of the 4^2^ full-factorial design for SIL-loaded cubosomes.

Formulation	P-407^a^ (% w/w)	PVA^a^ (% w/w)	EE (%)	Particle size (nm)	Q_8h_ (%)	PDI
C1	5	0	32.15 ± 2.05	446.02 ± 29.81	80.19 ± 3.16	0.21
C2	5	1	35.89 ± 1.13	357.92 ± 20.26	76.36 ± 4.32	0.10
C3	5	2.5	39.78 ± 2.29	308.55 ± 15.57	71.97 ± 3.38	0.15
C4	5	5	45.81 ± 1.75	320.34 ± 19.75	61.82 ± 2.86	0.18
C5	7.5	0	40.57 ± 2.45	252.20 ± 22.36	58.58 ± 3.36	0.13
C6	7.5	1	53.36 ± 2.03	236.60 ± 14.63	45.72 ± 3.06	0.19
C7	7.5	2.5	56.42 ± 3.24	210.04 ± 18.44	43.66 ± 2.35	0.08
C8	7.5	5	57.28 ± 2. 80	227.10 ± 9.11	37.53 ± 1.92	0.11
C9	10	0	43.84 ± 3.48	206.51 ± 10.19	52.88 ± 2.59	0.12
C10	10	1	46.28 ± 1.40	161.54 ± 6.24	48.48 ± 2.74	0.09
C11	10	2.5	65.89 ± 3.85	150.81 ± 6.86	44.08 ± 3.05	0.14
C12	10	5	66.47 ± 2.76	179.50 ± 9.75	31.85 ± 2.87	0.19
C13	15	0	57.34 ± 1.34	249.96 ± 13.10	29.09 ± 1.46	0.15
C14	15	1	59.63 ± 2.54	236.94 ± 18.27	25.74 ± 1.30	0.13
C15	15	2.5	67.82 ± 4.23	229.72 ± 11.39	24.12 ± 1.12	0.10
C16	15	5	72.01 ± 3.33	231.21 ± 17.22	21.03 ± 1.41	0.20

P-407: poloxamer 407; PVA: polyvinyl alcohol.

Concentration of glyceryl monooleate was 5% w/w with respect to the total weight of the dispersion.

Data are mean values (*n* = 3) ± SD.

a Concentration with respect to disperse phase.

### *In vitro* characterization of the prepared SIL-loaded cubosomes

#### Determination of SIL entrapment efficiency (EE%)

Samples from each formulation were centrifugated using cooling centrifuge (SIGMA 3-30K, Steinheim, Germany) at 14,000 rpm for 2 h at 4 °C for separation of the unentrapped drug. Aliquots from the separated cubosomes of each formulation were diluted using 1% w/v Triton-X-100 solution in phosphate buffer saline (PBS) pH 4.5 for disruption of any lipidic fragment (Rizwan et al., 2011; Aboud et al., 2016). The entrapped drug amount was calculated after analysis of the drug concentration in the diluted samples using UV spectrophotometer (Jasco, V-530, Tokyo, Japan) at a wavelength of 292 nm. SIL EE% was calculated using Equation (1):

(1) EE%= Entrapped drug/Total drug × 100

#### Determination of particle size

The mean particle size and polydispersity index (PDI) of SIL-loaded cubosomal formulations were assessed by dynamic light scattering (DLS) (Zetasizer Nano ZS, Malvern instruments, Malvern, UK). The samples were diluted with distilled water before measurement. All measurements were performed in triplicate at a temperature of 25 ± 2 °C and an angle of 90° to the incident beam (Aboud et al., 2016).

### *In vitro* release study of cubosomes

USP I dissolution apparatus (Hanson Research, SR 8 Plus model, Chatsworth, CA) adopting the membrane diffusion technique was used for measuring *in vitro* SIL release from the different cubosomal formulations. Cubosomal dispersion of different formulations (equivalent to 3 mg of SIL) was added to glass cylinders (6 cm length and 2.5 cm internal diameter) tightly covered from one end with the dialysis membrane with molecular weight cut-off of 12,000 Da, which was soaked in the receptor medium overnight. The loaded cylinders were fixed at the shafts of the USP dissolution tester apparatus (Abdelrahman et al., 2015). The release medium was 70 mL PBS pH 4.5 to mimic the pH of the vaginal environment. Rotation speed was adjusted to be 50 rpm with temperature set to 37 ± 0.5 °C throughout the dissolution study. At predetermined time intervals (0.5, 1, 2, 3, 4, 6 and 8 h), aliquots (1 mL) were withdrawn and the drug concentrations were spectrophotometrically analyzed at λ _max_ 292 nm. The release study was repeated three times and the average percentages release (±SD) were calculated using Equation (2):

(2) Drug release (%)= Mt/Mi × 100

where *M*_i_ and *M*_t_ are the initial amount of drug encapsulated in the cubosomes and the amount of drug released at time t, respectively (Aboud et al., 2016).

In order to analyze the release kinetics of SIL-loaded cubosomes, the obtained data were fitted into zero-order, first-order, Higuchi, Korsmeyer-Peppas, and Hixson–Crowell models. Selection of the appropriate mathematical model was based on the magnitude of the coefficients of determination (*R*^2^).

### Data analysis

The experimental design, checking the design fitting and adequacy, calculating the design parameter and studying the effect of each independent variable process parameters or their interactions on the dependent responses were generated by the Design Expert^®^ statistical program. The significance and validation of the chosen model were statistically assessed using the analysis of variance (ANOVA), *F* tests and correlation coefficients at a 95% significance level (*p* < .05). Moreover, a check point analysis was implemented to verify the reliability and validity of the generated mathematical model for dependent response predictions. The response surfaces were generated and the overlaying region of an overall desired response was corresponding to the optimum region where the cubosomal dispersions with appropriated properties can be obtained (Abdelrahman et al., 2015). Finally, desirability values were calculated and compared for the prediction of the formulation with the optimized characteristics. The optimized cubosomal formulation (OCF) was prepared in triplicates and compared the actual responses with the predicted ones according to the obtained regression equation.

### Transmission electron microscopy (TEM)

The morphology of the OCF was visualized using transmission electron microscope (JEM-1400, Jeol, Tokyo, Japan). One drop of diluted sample was placed on carbon-coated copper grid and stained by 2% w/v phosphotungistic acid (negative staining technique). The samples were investigated using TEM at 80 kV, after drying at room temperature (Mahmoud et al., 2017).

### Preparation of vaginal *in-situ* gelling sponges of SIL-loaded cubosomes

Chitosan (2% w/w) was incorporated into the optimized formulation of SIL-loaded cubosomes. The calculated amount of chitosan was sprinkled in the calculated amount of SIL-loaded cubosomes, with continuous stirring at room temperature until no lumps were observed and drops of 0.1 N acetic acid were needed to dissolve chitosan. The final concentration of SIL was 80 mg/mL. Aliquots (0.1 mL) were placed into blister molds and frozen at −25 °C for 1 h. The samples were then freeze-dried (0.25 mbar for 24 h with increasing shelf temperature −15 to 0 °C and a final drying for 2 h at +15 °C and 0.01 mbar) (Novalyphe-NL 500, Savant Instruments, Holbrook, NY, USA). The sponges were stored in desiccators over calcium chloride at room temperature until further use (Bertram & Bodmeier, 2006). For comparative purpose, free SIL *in situ* gelling sponges (FIS) were prepared adopting the same procedure (SIL concentration was 12.5 mg/mL in PBS pH 4.5).

### Histopathological study

#### Animal housing and handling

All experiments were approved by the Institutional Animal Ethics Committee, Beni-Suef University, Egypt. Forty female mature Wistar rats with an average weight of 200–250 g were obtained from the experimental animal care center, Faculty of Veterinary Medicine, Beni-Suef University. The animals were grouped randomly and housed in polyacrylic cages at a temperature of 22 ± 1 °C and relative humidity of 55 ± 5%. All animals had free accesses to normal chow and tap water *ad libitum*. The animals were kept in a dark:light cycle of 12 h each. Rats were left for seven days for adaptation before the beginning of the experiment. The female rats were divided into four groups of 10 rats each. The first group was used as a control while other groups received SIL with a dose of 16.6 mg/kg of body weight of rats either oral solution through oral gavage, intravaginal CIS, or intravaginal FIS for 14 days (Shanmugam et al., 2014).

#### Tissue collection and fixation

After 14 days by the end of the experiment, all rats were sacrificed and dissected carefully; different parts of the female genital tract (ovaries, uteruses, and vagina) were collected and then immersed in Bouin's fixative. Specimens from different parts of the female genital tract were dehydrated in ethyl alcohol, cleared in xylol, impregnated in soft paraffin, and embedded in hard paraffin. Sections of 4–6 µm were cut and mounted on clear and dry glass slides. The obtained slides were stained with Hematoxylin and Eosin (H & E) and Periodic acid Schiff (PAS) for histopathological examination (Bancroft & Gamble, 2008). Morphometric measurement of blood vessels in different parts of the female genital tract, endometrial thickening and vaginal epithelial thickening was performed by the aid of Image J analysis software program (NIH, Bethesda, MD), using LEICA (DFC290 HD system digital camera, Heerbrugg, Switzerland) connected to the light microscope using 10× objective lens.

### Pharmacokinetic studies

#### SIL administration to rats

Thirty female mature Wistar rats weighing 200-250 g obtained from the experimental animal care center, Faculty of Veterinary Medicine, Beni-Suef University, were divided into three groups each comprising 10 rats. SIL was administered in a dose of 16.6 mg/kg (Shanmugam et al., 2014). Group 1 received oral SIL solution (2.25 mg/ml) in purified water, group 2 received the optimized intravaginal CIS and group 3 received intravaginal FIS. Animals were allowed free access to food and water, until the night prior to dosing and were fasted for 10 h. Blood samples (1 ml) from retro-orbital plexus were collected at preset intervals of 0.5, 1, 2, 4, 6, 8 and 24 h. Blood samples were collected in heparinized tubes to avoid clotting and samples were centrifuged at 3000 rpm for 10 min to obtain the plasma.

The separated plasma tubes were stored at –20 °C until assayed. The protocol of this study was reviewed and approved by our institutional animal ethics committee of Beni-Suef University and all procedures for agent administration, blood and tissue collection were in accordance with the 8th edition of the Guide for the Care and Use of Laboratory Animals published in 2011 by the United States National Academy of Sciences.

#### Chromatographic conditions

The amount of SIL in the samples was estimated by adopting HPLC (Tripathi et al., 2013). The HPLC system (Agilent 1260 Infinity, Waldbronn, Germany), equipped with Agilent 1260 Infinity Diode array detector VL (G 1315D), Agilent 1260 Infinity preparative pump (G 1361A), Agilent 1260 Infinity thermostatted column compartment (G 1316A), and Agilent 1260 Infinity preparative Auto sampler (G 2260A). Separation and quantification were carried out on C18 column (ZORBAX Eclipse Plus, Agilent Technologies, Walnut Creek, CA, USA) (25 cm × 4.6 mm i.d., 5 µm particle size). The wavelength was set at 230 nm. The mobile phase was methanol:water (85:15 v/v) at a flow rate of 1 mL/min. The mobile phase was prepared daily and degassed by ultrasonication (Sonix TV ss-series, North Charleston, SC) before use.

#### Samples preparation for analysis

The frozen plasma samples were left to be thawed at room temperature. A liquid–liquid extraction procedure was used. Plasma samples (500 µL) were mixed with 1500 μL of acetonitrile and 5 mL of diethyl ether. After mixing for 30 s, the mixture was centrifuged for 10 min at 3000 rpm. The clear supernatant layer was transferred into another conical glass tube and organic layer completely evaporated at room temperature. After evaporation, the remaining residues were dissolved in mobile phase and 40 μL was injected using the autosampler.

#### Data analysis

Pharmacokinetic parameters were estimated from plasma data using computer program WinNonlin^®^ (version 1.5, Scientific Consulting, Inc., Rockville, MD, USA). Non-compartmental pharmacokinetic model was adopted for the calculation of the maximum drug concentration (*C*_max_, ng/mL) and time needed to reach this concentration (*T*_max_, h) from each rat plasma concentration–time curve. Trapezoidal rule method was adopted for calculation of the area under the curve (AUC) from 0 to 24 (AUC_0–24_, ng h/mL) and from 0 to infinity (AUC_0−∞_, ng h/mL).

#### Statistical analysis

All data were presented as mean ± SD. The results were analyzed statistically by one-way ANOVA with subsequent multiple comparisons using Tukey multiple comparisons test. The significance level was set at *p* < .05. All calculations were made using the computer program SPSS 22 (SPSS, Chicago, IL).

### Results and discussion

### Optimization of SIL-loaded cubosomes

#### Experimental design *via* (4^2^) full factorial

Design of experiments, one of the key elements of the quality by design principle, has been used for testing a large number of factors simultaneously and obviates the use of large number of independent runs when the traditional step-by-step approach is utilized (Badawi & El-Khordagui, 2014). Full factorial design (4^2^) is one of the prevalent methods in the design of experiments that includes the utilization of various kinds of experimental designs to create polynomial mathematical relationships and mapping the response over the experimental domain to choose the optimal process parameters (Shamma & Aburahma, 2014).

The intravaginal delivery of SIL is affected by the physiochemical properties of the nanocarriers. Accordingly, optimization of cubosomes was carried out using a (4^2^) full-factorial design based on chosen criteria. The effect of independent variables namely; P-407 and PVA concentrations on cubosomes EE% (*Y*_1_), particle size (*Y*_2_), and % SIL released from cubosomes in 8 h (*Y*_3_) were routinely evaluated to select their optimum levels. Responses of 16 cubosomal dispersions were listed and computed in Table 1 for assessing the selected parameters.

### Evaluation of SIL-loaded cubosomes

#### SIL entrapment efficiency

The EE% for the prepared SIL-loaded cubosomes ranged between 32.15 and 72.01%, Table 1. As shown in Table 1, SIL was successfully entrapped into the cubosomes which reveals the ability of lipid-based cubosomes as a delivery system for water and lipid soluble drugs. Statistical analysis using ANOVA showed that the sequential model proposed for assessing the calculated EE% values was polynomial quadratic model. Adequate precision was calculated by the Design-Expert software to demonstrate the signal–to-noise ratio, whereas a ratio greater than 4 is desirable indicating the validity of the utilized model to navigate the design space (de Lima et al., 2011; Ahmad et al., 2015). Also, predicted *R*^2^ was computed as a measure of how good the model could predict a response value by comparing the calculated value with the adjusted *R*^2^ (Annadurai et al., 2008). Adequate precision of 16.35 with a reasonable difference between the predicted *R*^2^ (0.8264) and the adjusted *R*^2^ (0.8794) was attained.

Accordingly, EE% values of SIL-loaded cubosomes were significantly affected by the two independent factors (*p* < .05). The regression equation relating the effect of P-407 (*X*_1_) and PVA (*X*_2_) concentrations on the EE% in terms of coded values is presented by Equation (3). From the regression coefficients, the concentration of both P-407 (*X*_1_) and PVA (*X*_2_) had a noticeable positive effect on EE% values.

(3) $$\text{EE}\%=61.62+\text{12}.{\text{46X}}_{\text{1}}+\text{8}.{\text{85X}}_{\text{2}}+0.{\text{79X}}_{\text{1}}{\text{X}}_{\text{2}}-\text{5}.\text{7}0{\text{X}}_{\text{1}}{}^{\text{2}}-\text{5}.0{\text{9 X}}_{\text{2}}{}^{\text{2}}$$

**Figure 1.**
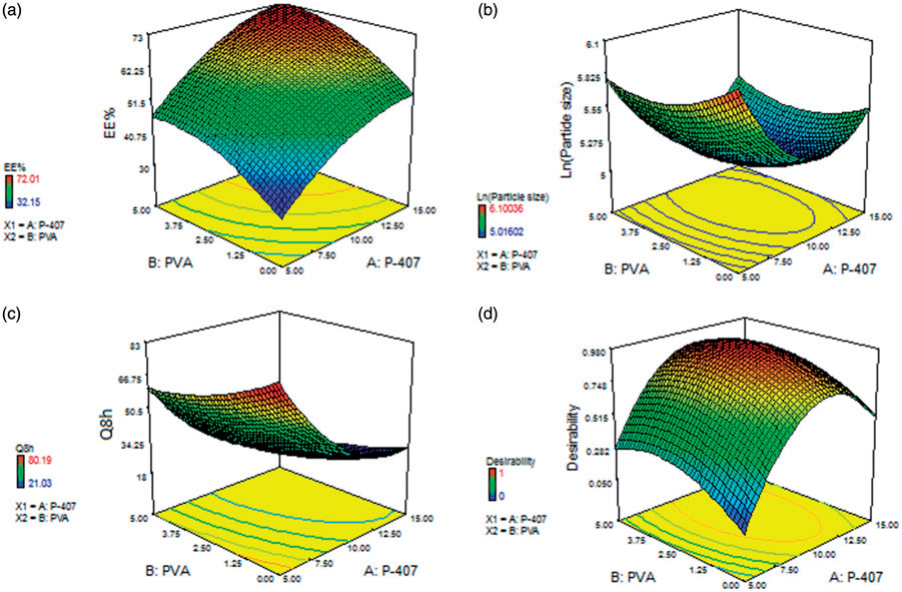
Response surface plot for the effect of P-407 (X1) and PVA (X2) concentrations on (a) EE%, (b) particle size, (c) Q8h, and (d) desirability of the prepared cubosomal dispersions.

The influence of P-407 (X_1_) and PVA (X_2_) concentrations on EE% is illustrated in Figure 1(a). The positive relationship of P-407 and PVA concentrations might be explained by their capability to provide a coat over the composed nanocubosomes to stabilize them. This composed coat is capable of holding extra drug, thus enhancing the EE% (Abdelrahman et al., 2015).

#### Particle size analysis of SIL-loaded cubosomes

Particle size assessment was carried out to emphasize that the prepared cubosomal dispersions are in nanometer range. It was reported that incorporation of 5% w/w disperse phase resulted in the formation of cubosomes with a monomodal dimensional distribution. Particle size of the formed cubosomal dispersions ranged between 150.81 and 446.02 nm as displayed in Table 1. PDI ranged between 0.08 and 0.21, which could be deemed as a reasonable mid-range (Cho et al., 2014). Particle size values were succumbed to polynomial analysis adopting a quadratic model. Factorial statistical analysis disclosed the ability of the mathematical model to explore the significance impact of P-407 (*X*_1_) and PVA (*X*_2_) concentrations on the cubosomes particle size. For fulfilling ANOVA assumption, the Box–Cox plot of particle size recommended logarithmic response transformation. The proposed model after transformation for particle size was with adjusted *R*^2^ of 0.9526, which was in a close conformity with the predicated one of 0.8787. The transformed regression equation [Equation (4)] depicting the response variation to the two factors in coded values was:

(4) $$\text{Ln}\left(\text{particle size}\right)=\text{5}.0\text{7}-0.\text{2}0{\text{ X}}_{\text{1}}-0.0{\text{74 X}}_{\text{2}}+0.0{\text{49 X}}_{\text{1}}{\text{X}}_{\text{2}}+0.{\text{49 X}}_{\text{1}}{}^{\text{2}}+0.{\text{16 X}}_{\text{2}}{}^{\text{2}}$$

The effect of P-407 and PVA concentrations on the cubosomal particle size is illustrated in Figure 1(b). These results demonstrated that the concentration of both P-407 and PVA had a significant influence on average particle size of cubosomes (*p* < .05). As shown in Figure 1(b), increasing the concentration of both P-407 and PVA from 5 to 10% and from 0 to 2.5%, respectively, resulted in a significant decrease in average particle size. On the contrary, the particle size increased significantly upon further increase in the concentration of both P-407 and PVA from 10 to 15% and from 2.5 to 5%, respectively. This result is in accordance with that reported by Morsi et al. (2014).

Indeed, GMO does not form a stable emulsion in water *per se* necessitating the incorporation of an emulsifier (Siekmann et al., 2002). It was reported that poloxamer dramatically stabilized vesicle state existing in dispersions of lipid (Almgrem et al., 2000). Particularly, P-407 was revealed to effectively enhance the stability of bicontinuous cubic phases or hexagonal dispersions. As reported from the phase diagram of GMO/P-407, P-407 is not solely adsorbed at surface of particles where its polypropylene oxide blocks are moored in the non-polar zone or at the GMO-based bilayers' surface; meanwhile, the polyethylene oxide tails are dissolved in the water (Nakano et al., 2001; Siekmann et al., 2002). This order promotes stability of the vesicles as a result of a robust steric repulsion among bilayers thereby diminishes their coalescence into bigger ones. Also, the resultant decrease in size with increasing P-407 concentration (up to 10%) might be due to the efficient decrease in interfacial tension between the lipid and water phase leading to the formation of smaller sized droplets. On the other hand, the large particle size obtained at the highest P-407 concentration (15%) could be attributed to disruption of GMO/P-407 ordering, therefore, decreasing the steric stability of the cubosomal dispersion (Morsi et al., 2014).

PVA is a polyol that is recognized as an efficient stabilizer and size controlling polymer which may be attributed to its ability to act as a dispersing agent that elevates the viscosity of the external water phase, thus enhancing emulsion stability (Baras et al., 2000). Also, it acts as a protective agent by being adsorbed at the lipid/aqueous interface; hence, lowers the fusion of the particles, decreases the aggregate formation, and minimizes the particle size. On the contrary, the significant increase in particle size at the highest PVA concentration (5%) may be due to increased viscosity of the polymer solution, leading to difficulty in dispersion, and subdivision of droplets (Patil et al., 2010). Elgindy et al. (2016) reported large cubosomal particle size with high PVA concentration which was attributed to deposition of PVA molecules onto the surface of cubosomes leading to increased particle size and PDI.

#### Release study of SIL from different cubosomal dispersions

The release profiles of SIL from the prepared cubosomal dispersions in PBS pH 4.5 are demonstrated in Figure S1 ((a and b), supplemental file). The *in vitro* release of SIL-based cubosomes showed a slight initial burst release followed by a sustained release over the experimental time. The observed slight initial burst release could be attributed to the dissolution of the adsorbed drug existing at or just below the cubosomal surface. While the followed sustained release was due to the entrapped SIL within the unique structured cubosomes. The % SIL release from cubosomes after 8 h was in the range of 21.03 to 80.19% as depicted in Table 1.

The proposed model was statistically significant in terms of residual analysis and ANOVA with adequacy/precision ratio of 26.69 manifesting adequate signal. Equation (5) denotes the quantitative effect of independent variables on the cubosomal release in coded values.

(5) $${\text{Q}}_{\text{8h}}=\text{28}.\text{46}-\text{22}.{\text{86X}}_{\text{1}}-\text{7}.{\text{99X}}_{\text{2}}+\text{2}.{\text{85X}}_{\text{1}}{\text{X}}_{\text{2}}+\text{15}.{\text{95X}}_{\text{1}}{}^{\text{2}}+\text{4}.{\text{51X}}_{\text{2}}{}^{\text{2}}$$

The examined independent variables pointed out a significant impact on the percentage of *in vitro* SIL release after 8 h between the different prepared formulations (*p* < .05), Figure 1(c). The noticed negative regression coefficients elucidated that raising P-407 and PVA concentrations had a negative effect on Q_8h_. It is clear that P-407 concentration revealed a predominant effect on Q_8h_.

It was observed that cubosome particles existed in Pn3 m (cD) patterns, which have sinuate pathway, at low concentration of P-407 and also most of the polymer adheres to cubic particles' surface while only few molecules are involved in the creation of the internal cubic phase structure (Rosen, 2005). On the other hand, when P-407 is incorporated at high concentration, it is embedded into the bulk of the matrix of the cubic phase with appearance of Im3 m (cP) patterns, which possess comparatively straight and short path. Expectedly, cubosomal particles with cD structure have higher drug release efficacy and the drug is more freely released than particles with cP structure (Zhao et al., 2004).

Meanwhile, increasing PVA concentration significantly decreased % SIL released as shown in Figure 1(c). A plausible explanation is that presence of PVA coat over the external surface of cubosomal particles that prolongs the diffusional path and hence, impeding SIL release rates (Gautam et al., 2007). Our release data revealed the capability of cubosomes to exhibit a sustained release manner which is a crucial matter for the fruitful design of drug delivery systems. A similar finding of oridonin release from GMO- and phytantriol-based cubosomes was reported by Shi et al. (2017).

Linear regression analysis of the release data elucidated that SIL was released from the cubosomal dispersions by a diffusion controlled mechanism, Table S1 (supplemental file). According to Nasr et al. (2015), cubosomes might be categorized as a burst release drug delivery systems, in which drug release is achieved by its diffusion from the matrix of the cubic phase. This result complies with previously reported findings (Morsi et al., 2014; Elgindy et al., 2016). Also, the mechanism of drug release from cubosomes was assessed employing the Korsmeyer–Peppas model. The calculated values of *n* among different formulations ranged from 0.53 to 0.71, indicating the non-Fickian diffusion of drug (0.45 < *n* < 0.89) and demonstrating anomalous release behavior where the diffusion may be combined with swelling of GMO bilayers (Elgindy et al., 2016; El-Nabarawi et al., 2016).

### Check point analysis

#### Validation of the statistical model

Upon comparing the values of the adjusted *R^2^* with the predicted ones, they were in a reasonable accordance indicating the significance and predictive capability of the factorial design. Moreover, the acceptable residuals and actual/predicted ratios with a small percentage error (<10%) were noticed between the actual and predicated response variables, evidencing the lack of curvature in the responses and validity of the factorial model (Badawi & El-Khordagui, 2014).

#### Selection of the OCF

The desirability was computed by Design-Expert software for optimization of the investigated responses based on the obtained results. Simultaneous optimization of all studied factors was not possible because the optimal result observed with one response may be accompanied with the worst condition for the other one in the same formulation (Abdelrahman et al., 2015). Desirability was determined to prognosticate the optimum composition with the maximal EE and Q_8h_ and the minimal particle size. Higher priority for optimization was offered to particle size and Q_8h_ over EE%. The predicated OCF was recognized, prepared in triplicates, and estimated for the dependent responses.

Regarding the validity of factorial design to prognosticate variable-dependent responses of the OCF, a check point analysis was applied for the comparison of the actual experimental values with predicted ones and a small % bias was observed which ranged from 1.21 to 3.43% for various responses. Our results emphasize the fitting and adequacy of the applied mathematical model for dependent responses prognosis.

The highest desirability value was found to be 0.978, as illustrated in Figure 1(d). This desirability value was for the cubosomal formulation containing 10.95% P-407 and 3% PVA. Thus, this formulation was picked for subsequent investigations.

#### Morphology of SIL-loaded cubosomes

**Figure 2.**
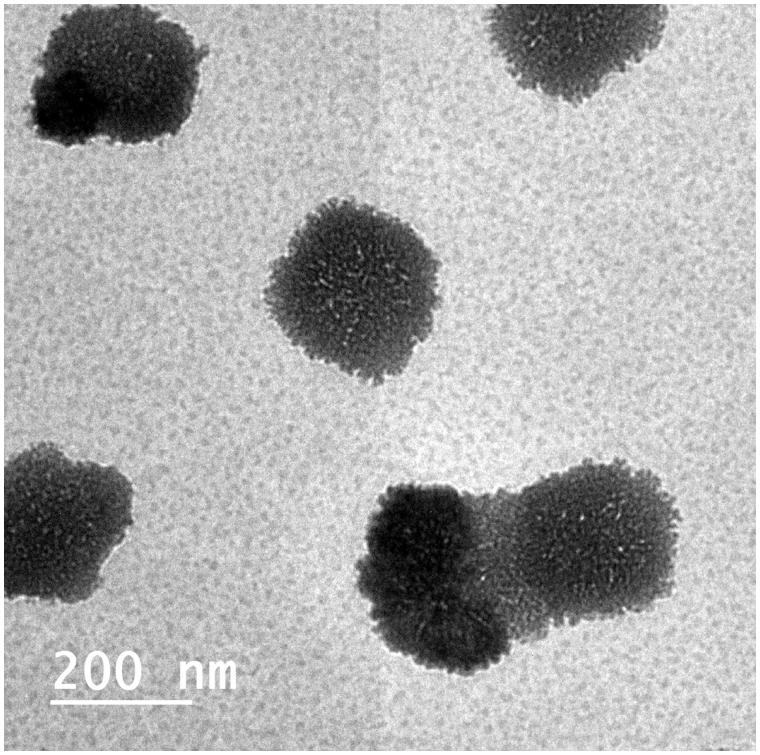
Transmission electron micrograph of the optimized cubosomal formulation (OCF).

The morphology of the OCF was examined using TEM, as shown in Figure 2. The transmission electron micrograph shows that the prepared cubosomes are nano-sized and in harmony with particles size measured with DLS. The micrograph shows that the particles are cubic in shape, uniform in size, well dispersed and well separated from each other.

#### Fabrication of vaginal *in-situ* gelling sponges

Preliminary studies were accomplished to determine the appropriate concentration of chitosan (1, 2, and 3% w/w) before selection of the final concentration incorporated into the optimized cubosomal dispersion. Optimization was considered to select the adequate concentration by measuring mucoadhesive potential, water uptake, mechanical properties, and *in vitro* drug release from the sponges. Sponges containing 1% w/w chitosan were delicate and showed low hardness of 1.11 N where a hardness of approximately ≥2 N is necessary to allow easiness of handling without damage (Bertram & Bodmeier, 2006) while 3% w/w chitosan-based sponges showed a significantly retarded drug release pattern. *In situ* gelling sponges containing 2% w/w chitosan was chosen for further investigations as they were hard enough to be easily removed from their blister packaging and at the same time still elastic with high extent of water uptake, extended drug release, and reasonable mucoadhesive strength that could retain the drug within the vaginal cavity (Table S2, Figures S2 and S3, supplemental file).

**Table 2.** Morphometric measurement of vaginal tissues in different studied groups.

		Diameter of large blood vessel			
Group	Endometrial thickening	Ovarian	Uterine	Vaginal	Vaginal epithelial thickening
Normal control	1013.70 ± 30.14	77.79 ± 6.85	137.50 ± 18.67	36.39 ± 4.80	131.33 ± 9.63
Oral SIL solution	1246.20 ± 30.06^a^	185.15 ± 20.582^a^	185.64 ± 40.94^a^	194.50 ± 10.16^a^	166.66 ± 10.17^a^
Intravaginal CIS	1381.70 ± 29.61^a^^,^^b^	206.34 ± 19.23^a^^,^^b^	234.20 ± 69.40^a^^,^^b^	198.03 ± 13.16^a^^,^^b^	183.32 ± 5.16^a^^,^^b^
Intravaginal FIS	1105.20 ± 33.32^a^^,^^b,c^	112.46 ± 10.33^a^^,^^b,c^	162.32 ± 22.21^a^^,^^b,c^	142.46 ± 9.43^a^^,^^b,c^	142.42 ± 7.87^a^^,^^b,c^

CIS: cubosomal *in situ* gelling sponges; FIS: free SIL *in situ* gelling sponges.

Values are means ± SD, with the number of animals =10 for each group.

Using one-way ANOVA followed by Tukey’s post-hoc test.

a *p* < .05 versus normal control.

b *p* < .05 versus oral SIL solution.

c *p* < .05 versus intravaginal CIS.

#### Histopathological study

**Figure 3.**
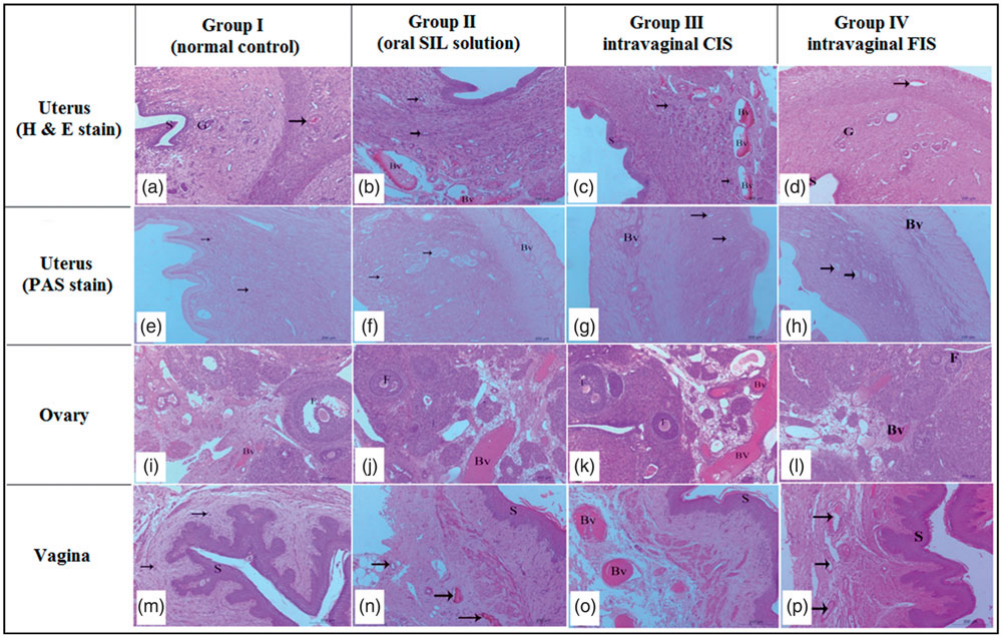
Histopathological characteristics of uterus, ovary and vagina from different studied groups after two weeks of SIL treatment. Hematoxylin and Eosin (H & E) staining. a–d: uterus of female Wistar rats. Magnification** **×100. (a) Uterus of normal group showing endometrium lined by simple columnar epithelium (S), normal endometrial gland (G) and blood vessels (arrow). (b) Uterus of rat in oral-treated group showing increased height of endometrial epithelium, activity of endometrial glands (arrow), and increased vascularity of uterus (Bv). (c) Uterus of rat in intravaginal CIS-treated group showing a massive increase in the endometrial epithelium (S), endometrial glands (G) and number of blood vessels as well as the blood flow (Bv). d- uterus of rat in intravaginal FIS-treated group showing the endometrial epithelium (S) and endometrial glands (G) similar to normal group with minor increase in number of blood vessels (arrow). (e–h): uterus of female Wistar rats. Periodic acid Schiff (PAS) staining. Magnification** **×100. (e) Uterus of normal group showing moderate reaction in the endometrial glands (arrow). (f) Uterus of rat in oral-treated group showing increased activity of endometrial glands (arrow). Note, increased vascularity of uterus (Bv). (g) Uterus of rat in intravaginal CIS-treated group showing a massive increase in the endometrial vascularity (Bv). Note, strong reaction in the endometrial glands (arrow). (h) Uterus of rat in intravaginal FIS-treated group showing normal endometrial vascularity (Bv) with moderate reaction in the endometrial glands (arrow). (i–l) Ovary of female Wistar rats. Magnification** **×100. (i) Ovary of rat in normal group showing normal ovarian tissue with growing follicles (F) and normal ovarian blood vessels (Bv). (j) Ovary of rat in oral-treated group showing increased vascularity of ovarian tissue (Bv) and growing follicles (F). (k) Ovary of rat in intravaginal CIS-treated group showing a massive increase in the ovarian tissue vascularity (Bv) and growing follicles (F). (l) Ovary of rat in intravaginal FIS-treated group showing a slight increase in the ovarian tissue vascularity (Bv) and growing follicles (F). (m–p): Vagina of female Wistar rats. (m) Vagina of normal group showing normal vaginal epithelium (stratified squamous epithelium less keratinized (S)) and normal vaginal blood vessels (arrow). (n) Vagina of oral-treated group showing increased height of vaginal epithelium (S) and increased vascularity of vaginal tissue (arrow). (o)- vagina of intravaginal CIS-treated group showing a massive increase in height of vaginal epithelium (S) and increased vascularity of vaginal tissue (Bv). (p) Vagina of rat in intravaginal FIS-treated group showing increased height of vaginal epithelium (S) and minor changes in vascularity of vaginal tissue (arrow).

Figure 3(a,e) demonstrates the normal histological architecture of uterine layers (endometrium, myometrium, and perimetrium) of normal rat (group 1, control); the endometrium appeared lined with simple columnar epithelium overlaying fibroelastic connective tissue of submucosa housing endometrial glands with active secretory columnar epithelium. The submucosa was housing blood vessels with different sizes. The oral SIL-treated group (group 2) exhibited increased uterus vascularity (Figure 3 (b and f)), while a slight increase in uterine vascularity was observed in intravaginal FIS-treated group (group 4) (Figure 3 (d and h)). Interestingly, intravaginal CIS-treated group (group 3) showed a massive increase in the endometrial epithelium, endometrial glands, and number of blood vessels as well as the blood flow (3 (c and g)). Additionally, an increase in the thickness of the endometrial layer from 1013.70 ± 30.14 µm (control group) to 1381.70 ± 29.61 µm which was significant compared with control, intravaginal FIS or oral-treated groups (Table 2). Moreover, angiogenesis was found only in the intravaginal CIS-treated group. These findings emphasize the increased availability of the CIS to endometrial tissue. Decreased vaginal SIL permeation was reported as it exists in cationic state and lactic acid produced by normal vaginal microflora results in acidified vaginal secretions (pH 3.5–4.5) leading to a significant decrease in drug permeation (Boskey et al., 2001; Wang et al., 2008).

Nevertheless, the incorporation of SIL into cubosomes significantly ameliorated its efficacy which might be attributed to enhanced permeability due to nanocarrier formulation, which acts as a penetration enhancer due to the dual effect of hydrophobic and hydrophilic domains in addition to the impact of GMO as a permeation enhancer. Also, cubosomes are good candidates for vaginal drug delivery as these systems are mucoadhesive since their components have hydroxyl groups that enable hydrogen bonding with mucus membranes (Garti et al., 2012; Abdelrahman et al., 2015). Moreover, large surface area and good fluidity of cubosomes allow intimate contact with mucous membranes.

Additionally, this finding could be attributed to the spongy structure of the formulation which is a crucial factor to ascertain fast hydration and gelation at the vaginal mucosa leading to a larger contact/surface area and boosted the water uptake by capillary forces. The intensive bioadhesive characteristics of chitosan, the ingredient of the *in situ* gelling sponge, elongate the residence time of cubosomes on endometrial surface and so, lower the vaginal drainage. Chitosan exhibits its mucoadhesive properties through interaction between its positively charged amino groups with the negatively charged sialic acid residues of the glycoproteins of the mucosal epithelial cells. Also, chitosan adopts a more elongated shape, assumes high charge density and becomes more ionized at low pH values like vaginal pH resulting in tighter contact and enhanced electrostatic interaction with sialic acid. Moreover, it exhibits permeation enhancing capability *via* amelioration of the paracellular transport mechanism (Valenta, 2005).

#### Pharmacokinetic studies

Table 3 shows the pharmacokinetic parameters in rat plasma after administration of oral SIL solution, intravaginal CIS, and intravaginal FIS. SIL dose for the three treatments was fixed at 16.6 mg/kg of body weight of animals. Both oral and intravaginal administrations were highly tolerable by the rats.

**Table 3.** Mean pharmacokinetic parameters for SIL in rat plasma following administration of oral solution, intravaginal CIS, and intravaginal FIS.

Pharmacokinetic parameter	Mean ± SD		
	Oral solution	Intravaginal CIS	Intravaginal FIS
*C*_max_ (ng/mL)	1283.37 ± 219.11	134.30 ± 21.21^a^	74.20 ± 12.35^a^^,b^
*t*_max_ (h)	0.88 ± 0.29	8.50 ± 1.00^a^	4.55 ± 0.43^a^^,b^
*K*_elim_ (h^−1^)	0.1641 ± 0.0326	0.0865 ± 0.0111^a^	0.1298 ± 0.0154^a^^,b^
*t*_1/2_ (h)	4.22 ± 0.51	8.02 ± 0.88^a^	5.34 ± 0.37^b^
AUC_0–24_ (ng.h/mL)	6556.54 ± 844.29	1544.86 ± 389.48^a^	747.76 ± 211.68^a^^,b^
AUC_0–∞_ (ng.h/mL)	6655.93 ± 837.19	2012.56 ± 295.57^a^	1035.65 ± 223.59^a^^,b^
*F*_rel_ (%)	–	30.24 ± 10.32	15.56 ± 5.86^b^

CIS: cubosomal *in situ* gelling sponges; FIS: free SIL *in situ* gelling sponges.

Values are means ± SD, with the number of animals =10 for each group.

Using one-way ANOVA followed by Tukey’s *post-hoc* test.

a *p* < .05 versus oral SIL solution.

b *p* < .05 versus intravaginal CIS.

The AUC_0−∞_ and *C*_max_ of SIL in plasma were 6655.93 ± 837.19 ng h/mL and 1283.37 ± 219.11 ng/mL, 2012.56 ± 295.57 ng h/mL, and 134.3 ± 21.21 ng/mL and 1035.65 ± 223.59 ng h/mL and 74.20 ± 12.35 ng/mL, following administration of oral, intravaginal CIS, and intravaginal FIS, respectively. The oral administration exhibited nearly 3- and 6-fold increase in the AUC_0–∞_ and exhibited nearly 10- and 17-fold increase in the *C*_max_ compared to both intravaginal CIS and intravaginal FIS, respectively.

The *T*_max_ of oral, intravaginal CIS, and intravaginal FIS administrations were 0.88 ± 0.29, 8.5 ± 1 and 4.55 ± 0.43 h, respectively. The *t*_1/2_ was 4.22 ± 0.51 h for oral administration, while it was 8.02 ± 0.88 h and 5.34 ± 0.37 h for intravaginal CIS, and intravaginal FIS applications, respectively. A sustained and slow SIL absorption *via* vaginal mucosa was elucidated by the retarded *T*_max_ and prolonged *t*_1/2_. Compared to the oral solution, the % relative bioavailability of SIL from the intravaginal CIS was about 30.24% and it was 15.56% for intravaginal FIS. As being intended for local vaginal administration, the lower SIL plasma concentrations from both intravaginal CIS and intravaginal FIS are robustly beneficial because high systemic exposure was documented to make different unpleasant adverse effects including headaches, flushing, and hypotension (Alexander et al., 2004; Shanmugam et al., 2014). Meanwhile, intravaginal FIS exhibited minor uterine absorption as a result of diminished SIL permeability at uterine pH as mentioned previously.

The vaginal route has been stated to possess the capability for uterine targeting of drugs like danazol and progesterone (De Ziegler et al., 1997; Cicinelli et al., 1998). Intravaginal administered progesterone exhibited a lower plasma concentration in the radial artery than in the uterine artery pointing out a preferential uterine progesterone distribution and emphasizing the presence of direct local transfer from vagina to uterus known as first uterine pass effect (De Ziegler et al., 1997; Cicinelli et al., 1998). Our findings of the CIS well comply with these previous results proposing the potential intervention of first uterine pass effect of SIL following intravaginal application. The counter-current transport from vaginal veins to the uterine artery is one of the suggested mechanisms which elucidates the preferable distribution to the uterus following intravaginal application (Taurin et al., 2017; Gomaa et al., 2018). Our study indicated that intravaginal administration of CIS for uterine SIL targeting was more beneficial than both intravaginal FIS and oral administration and could be considered as a promising approach where increased uterine exposure (as demonstrated in histopathological study) coupled with decreased systemic levels were attained.

The biodistribution study measuring SIL concentration in uterus may represent a limitation to our study. Biodistribution study can confirm the results of histopathological and pharmacokinetic studies.

## Conclusions

In this study, SIL-loaded nanocubosomes were developed using GMO, P-407, and PVA. The OCF containing 10.95% P-407 and 3% PVA showed the smallest particle size, high EE, and a sustained release profile. Chitosan (2% w/w) was incorporated into the OCF and then lyophilized into small sponges. Intravaginal administration of the CIS revealed a significant increase in the endometrial thickness with congestion and dilatation of the endometrial blood vessels compared to either intravaginal FIS or oral-treated groups. The *in vivo* pharmacokinetic study demonstrated significantly lower *C*_max_ and AUC_0–∞_ of SIL in plasma with a prolonged mean elimination half-life following intravaginal administration of the CIS compared to oral administration of SIL solution. Our investigation proposed the potential intervention of first uterine pass effect of SIL after intravaginal administration. Based upon the foregoing findings, the CIS promoted SIL uterine exposure after intravaginal application and hence, could be used for beneficial uterine targeting.

## Supplementary Material

Supplemental Material
