# Supplementary material for: Novel in situ gelling vaginal sponges of sildenafil citrate-based cubosomes for uterine targeting
Source: Drug Deliv. 2018 Jun 5;25(1):1328–39. doi: 10.1080/10717544.2018.1477858 (PMC6058503; doi:10.1080/10717544.2018.1477858)
Supplement: Supplemental Material [file IDRD_A_1477858_SM6324.docx]

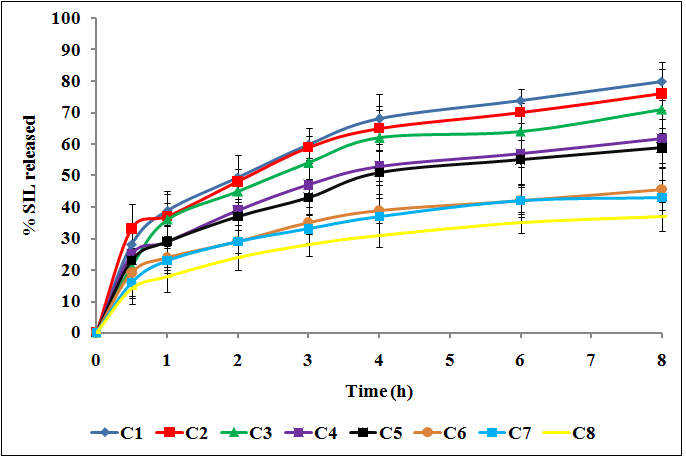

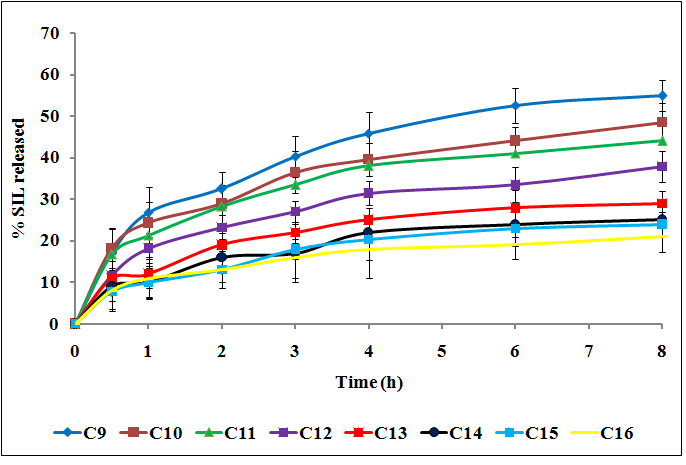
**Fig. S1**: *In vitro* release profile of SIL from different cubosomal formulations: (a) C1-C8 and (b) C9-C16.

**(b)**

**(a)**

Table (S1): Kinetic parameters of SIL release from the cubosomal dispersions

| Formulation | Zero | First | Diffusion | Mechanism |
| --- | --- | --- | --- | --- |
|  | R^2^ | | |  |
| C1 | 0.9186 | 0.9018 | 0.9278 | Diffusion |
| C2 | 0.8840 | 0.8740 | 0.9712 | Diffusion |
| C3 | 0.9319 | 0.9330 | 0.9876 | Diffusion |
| C4 | 0.8709 | 0.8379 | 0.9748 | Diffusion |
| C5 | 0.8529 | 0.8321 | 0.9659 | Diffusion |
| C6 | 0.9650 | 0.9416 | 0.9854 | Diffusion |
| C7 | 0.8652 | 0.8677 | 0.9778 | Diffusion |
| C8 | 0.9917 | 0.9568 | 0.9967 | Diffusion |
| C9 | 0.8976 | 0.8566 | 0.9942 | Diffusion |
| C10 | 0.9229 | 0.8211 | 0.9853 | Diffusion |
| C11 | 0.9786 | 0.9513 | 0.9829 | Diffusion |
| C12 | 0.9643 | 0.9465 | 0.9877 | Diffusion |
| C13 | 0.9243 | 0.9188 | 0.9397 | Diffusion |
| C14 | 0.9252 | 0.9446 | 0.9763 | Diffusion |
| C15 | 0.9454 | 0.9634 | 0.9851 | Diffusion |
| C16 | 0.9085 | 0.9110 | 0.9278 | Diffusion |

Table (S2): Hardness and mucoadhesive strength of sponges prepared from different chitosan concentrations

| Chitosan concentration  (% w/w) | Hardness  (N) | Mucoadhesive strength  (dyne/cm^2^) |
| --- | --- | --- |
| 1 | 1.11 ± 0.23 | 2899.76 ± 71.16 |
| 2 | 2.10 ± 0.12 | 3567.59 ± 80.02 |
| 3 | 3.00 ± 0.30 | 4373.66 ± 66.59 |


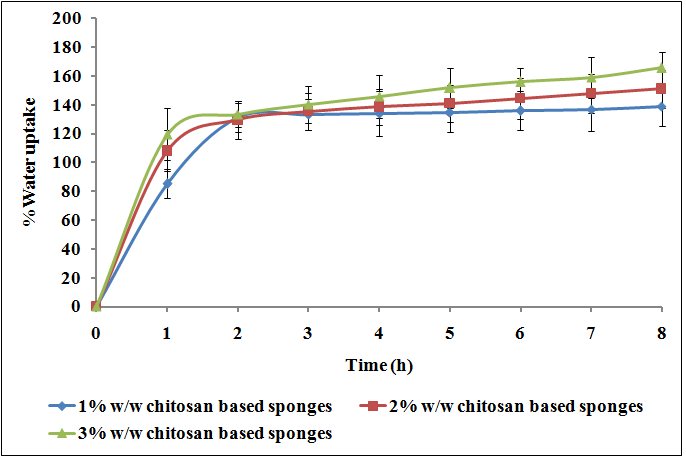


Fig. S2: Water uptake of sponges prepared from different chitosan concentrations.


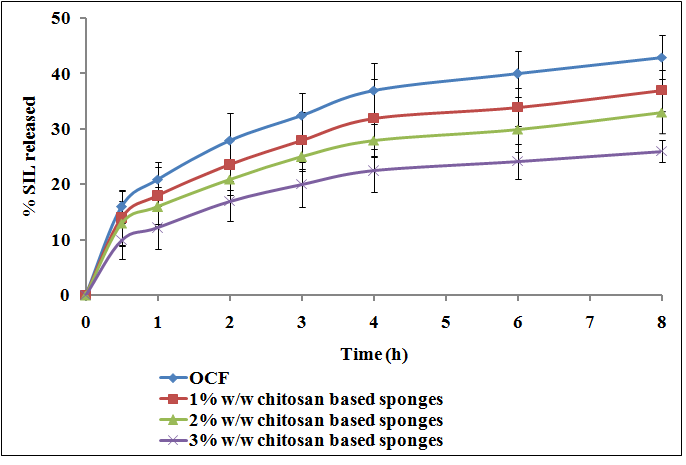


Fig. S3: *In vitro* release profile of SIL from the prepared cubosomal *in situ* gelling sponges in comparison with the optimized cubosomal formulation (OCF).
